# Desulfurization of Model Oil by Selective Adsorption over Porous Boron Nitride Fibers with Tailored Microstructures

**DOI:** 10.1038/s41598-017-03600-4

**Published:** 2017-06-12

**Authors:** Zhiyi Yan, Jing Lin, Xiaohai Yuan, Tao Song, Chao Yu, Zhenya Liu, Xin He, Jianli Liang, Chengchun Tang, Yang Huang

**Affiliations:** 1 School of Materials Science and Engineering, Hebei University of Technology, Tianjin, 300130 P.R. China; 2 Hebei Key Laboratory of Boron Nitride Micro and Nano Materials, Hebei University of Technology, Tianjin, 300130 P.R. China

## Abstract

We report on the controllable synthesis of porous BN microfibers and explore their applications as adsorbent for removing dibenzothiophene (DBT) in model oil. The growth evolution of porous BN microfibers has been carefully investigated by correlating their structural characteristics with their synthesis conditions. The as-prepared BN microfibers exhibit very high adsorption capacity for DBT (86 mg S g^−1^ according to the Langmuir isotherm model), showing excellent adsorptive desulfurization performance. The porous BN after adsorption can be regenerated by a simply heat treatment. After four times recycling, the regenerated adsorption capacity still remains more than 83% of that at the first adsorption. The superb oxidation resistance and chemical inertness, high sulfur adsorption capacity, as well as excellent regeneration performance render the developed porous BN microfibers to be a decent adsorbent for sulfur removal from fuels.

## Introduction

Hexagonal boron nitride (*h*BN) has gained much interests because of its structural analogue to the counterparts of carbon and its unique combination properties including low density, electrical insulation, high thermal conductivity, superb oxidation resistance and chemical inertness, etc.^1–4^. The properties of BN could be tailored with rational designed morphology, microstructure and surface chemistry, thus find many functional applications beyond those of traditional refractory materials^5–11^. For example, porous BN with high specific surface areas has recently been intensively studied as a novel absorbent for promising applications including hydrogen storage, water treatment, etc.^12–25^. Especially, porous BN micro/nanostructures, such as porous nanofibers^17, 25^, porous nanosheets^15^, not only hold most of the features and advantages of BN micro/nanostructures, but also possess much larger specific surface areas and more active reaction sites. These attractive features make them display excellent adsorption performances for metal ions and organic pollutants.

Very recently, the researches on porous BN as adsorbent for sulfur removal from fuels have attracted increasing attention^26–29^. Porous BN materials have demonstrated attractive adsorptive desulfurization performance due to their high specific surface areas and large pore volumes. Besides, compared with traditional adsorbents, such as activated carbon, zeolites, and mesoporous silica, the superb oxidation resistance, thermal stability and chemical inertness of porous BN imply their superior regeneration performance and potential applications in harsh environment. Moreover, the polar B-N bonds are beneficial for the selective desulfurization. Therefore, porous BN can be considered as a promising adsorbent for desulfurization from fuels.

As the adsorbent properties of porous BN can depend strongly upon their detailed microstructure, e.g. specific surface areas, pore sizes, as well as lattice defects, the controllable synthesis of porous BN with tailored microstructure is of great importance for exploring their desulfurization. In our earlier studies, we have successfully synthesized porous BN microfibers via a two-step method using melamine and boric acid as source materials^18^. The porous BN in fibrous morphology could not only do as adsorbents in powder form but also be made into more applicative fibrous assemblies e.g. filtration membranes, benefiting from their high aspect ratios^30^. Herein, we report on the controllable synthesis of porous BN microfibers and explore their potential applications as adsorbent for desulfurization. Firstly, the evolution of the porous BN microfibers was carefully investigated by correlating their determined structural characteristics with their synthesis conditions. Then the BN microfibers obtained under different synthesis conditions were used as adsorbent for removing dibenzothiophene (DBT) in model oil. Their adsorption capacities were evaluated and the effects of microstructures of porous BN on their adsorption properties were studied in detail. Finally, the porous BN microfibers after adsorption were regenerated by a simply heat treatment and their recycle performances were evaluated.

## Results and Discussion

The synthesis of porous BN microfibers includes two steps. Firstly, C_3_N_6_H_6_·2H_3_BO_3_ (M·2B) precursor was prepared through the precipitation of hot H_3_BO_3_ and C_3_N_6_H_6_ solution. Then porous BN microfiber was obtained via the decomposition of M·2B precursor. The formation reaction can be expressed as 2H_3_BO_3_ + C_3_N_6_H_6_ → C_3_N_6_H_6_·2H_3_BO_3_ → 2BN + 3H_2_O + 3CO + 2NH_3_ + N_2_. Detailed growth mechanism has been discussed in our previous work^18^. Herein we mainly focus on the evolution study of the porous BN by correlating their microstructure with synthesis conditions. A series of porous BN microfibers were prepared via the calcination of M·2B precursor at different temperatures (900 °C, 1000 °C, 1100 °C, 1200 °C, 1300 °C and 1400 °C), and their structures were characterized by X-ray powder diffraction (XRD), Fourier transform infrared (FTIR) spectrophotometer and Raman spectrophotometer.

**Figure 1:**
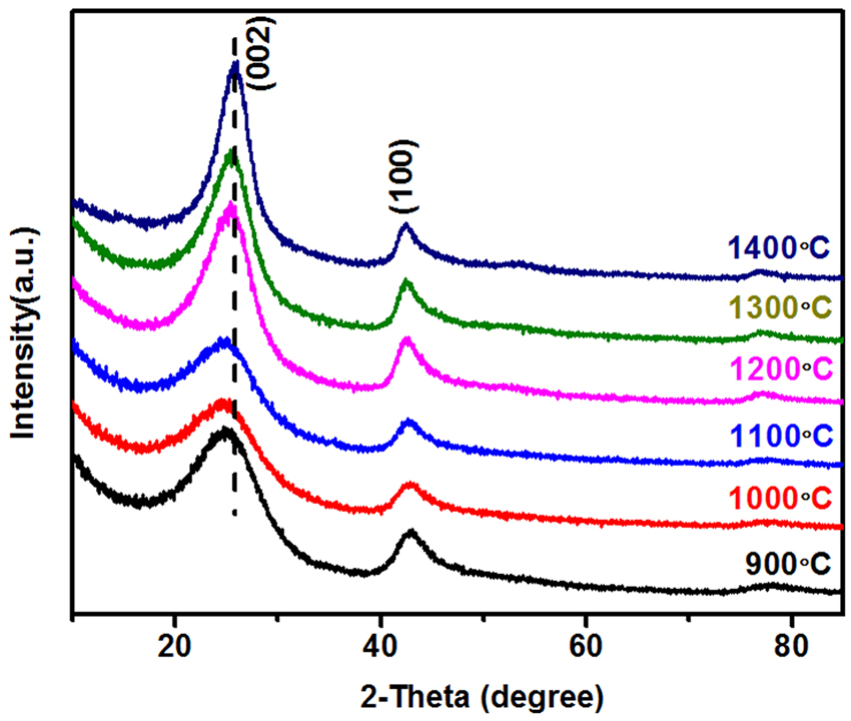
XRD patterns of porous BN microfibers prepared at different calcination temperatures.

In general, the yields of the BN products obtained under different calcination temperatures (from 900 °C to 1400 °C) are all around 100% (Table S1, Supporting Information), indicating that the transformation from M·2B precursors to BN has almost been completed even at 900 °C. All the BN products show as snow-white powders as shown in Fig. S1. XRD patterns of the as-prepared samples are displayed in Fig. 1. All of them display diffraction peaks at 2*θ* = 24.69 ~ 25.98° and 2*θ* = 42.75 ~ 42.39°, corresponding to the (002) and (100) plane spacing of *h*BN. With increasing of calcination temperatures, the (002) diffraction peaks shift to higher angles, and the peak intensities also increase gradually, indicating that the porous BN has a higher degree of crystallinity after pyrolysis at increased temperatures.

**Figure 2:**
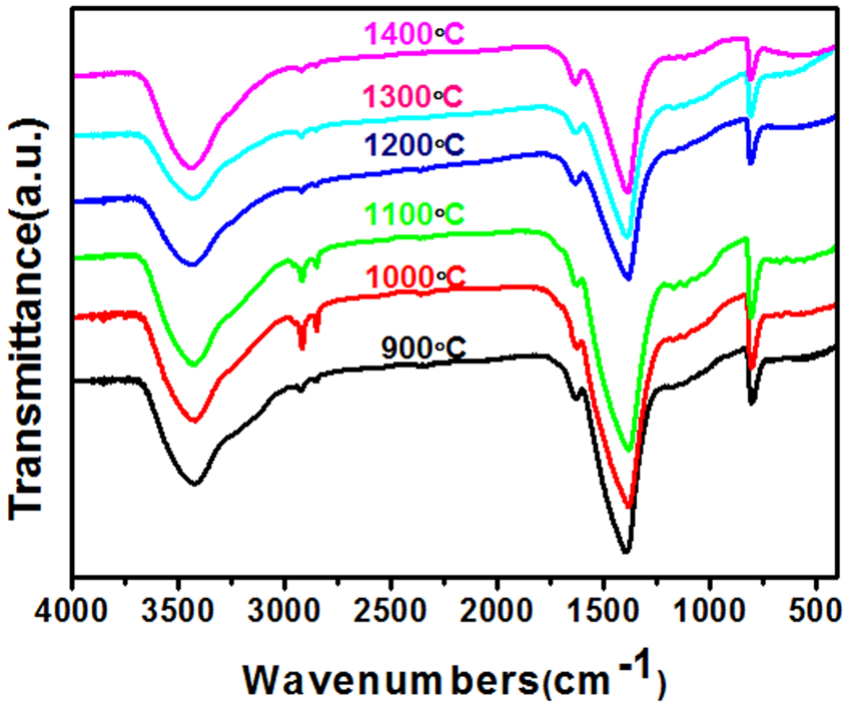
FTIR spectra of porous BN microfibers prepared at different calcination temperatures.

Figure 2 shows FTIR spectra of the different BN fibers. It can be seen that two main peaks at ~1400 and ~800 cm^−1^ correspond to the B–N stretching vibrations and B–N–B bending vibrations^11, 19, 31–33^, respectively. Both of the two main peaks can be detected in all of the BN samples, which implies the formation of a BN phase, even at a low calcination temperature of 900 °C. The broad vibration bands at ~3420 cm^−1^ should derive from the O-H stretching vibration mode from sp^3^-hyridized N_3_B(OH) units, and the shoulder peaks at ~3240 cm^−1^ can be ascribed to the N-H stretching vibration. Besides, additional surface bonds, such as C–H (~2964, ~2923, ~2852 and ~1265 cm^−1^), B–N–O (~1100 and ~920 cm^−1^) and N–H (~1630 cm^−1^), are also observed in all of the porous BN samples. FTIR results indicate that all the as-prepared BN samples contain C-, O- and H-related impurities^11, 32, 33^. We also note that the relative intensities of the peaks deviated from non B-N bonds decreased with increased calcination temperatures in general, which means that the number of the impurity atoms were reduced in the BN microfibers synthesized at higher calcination temperature.

**Figure 3:**
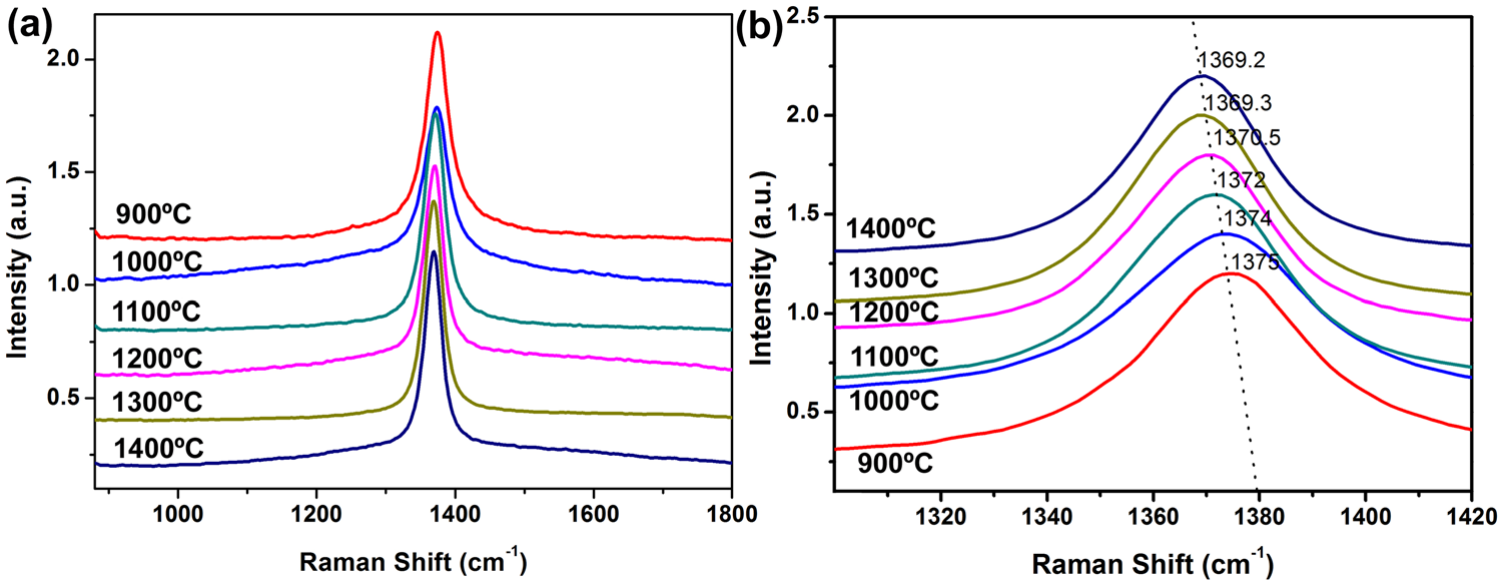
Raman spectra of porous BN microfibers prepared at different calcination temperatures.

The Raman spectra of the as-synthesized samples are shown in Fig. 3. All the spectra have a strong peak at ~1370 cm^−1^, corresponding to the counter-phase B-N E_2g_ vibration mode within BN sheets. Moreover, with increasing calcination temperature from 900 to 1400 °C, the peak gradually shifts from 1375 cm^−1^ to 1368 cm^−1^, further indicating that BN fibers prepared at low synthesis temperature have a weak interlayer interactions.

**Figure 4:**
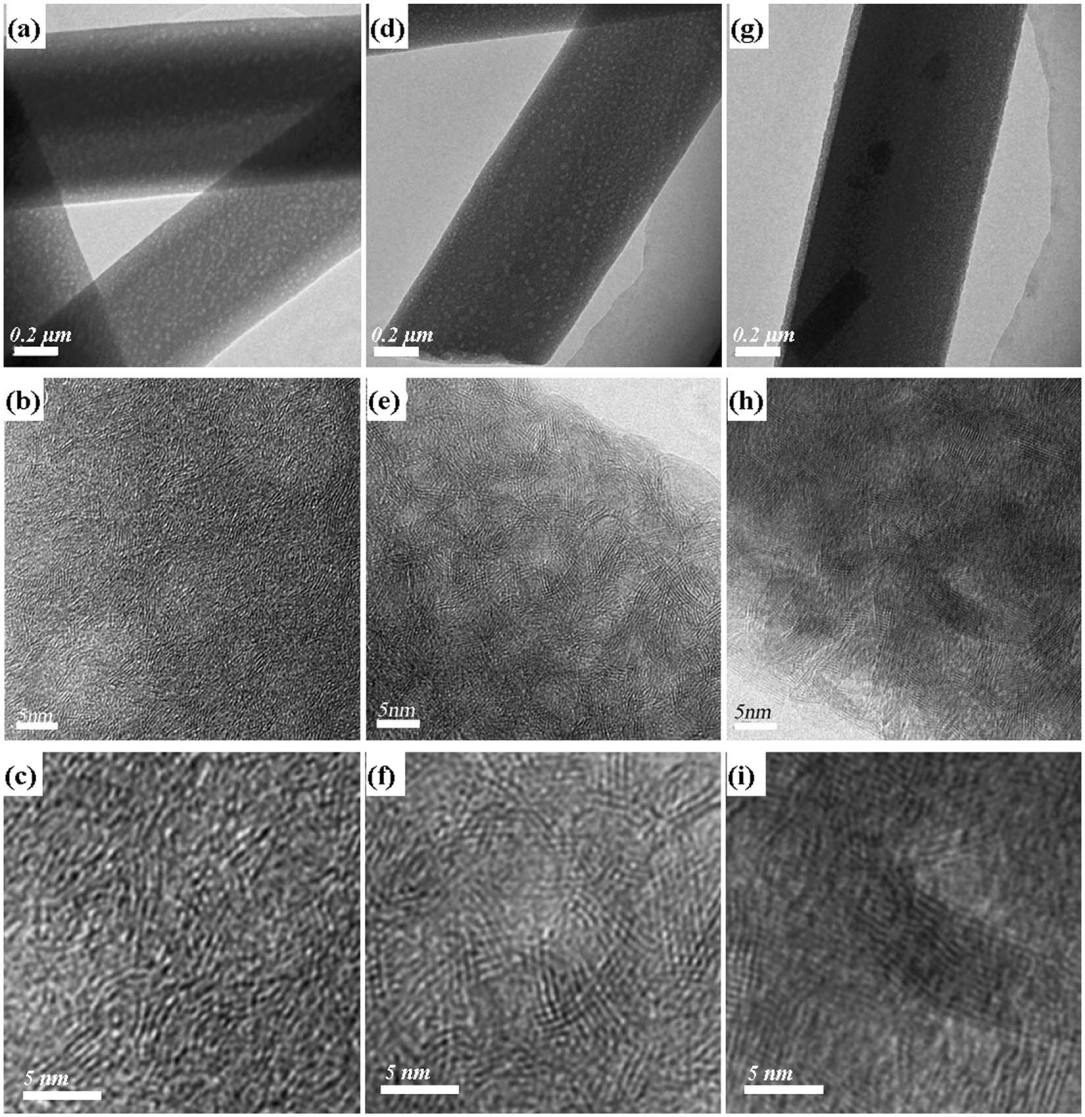
TEM images of porous BN microfibers prepared at 900 °C (**a**–**c**), 1100 °C (**d**–**f**) and 1400 °C (**g**–**i**).

Then transmission electron microscopy (TEM) was used to further analyze the structures of porous BN fibers in detail. Figure 4 displays typical TEM images of porous BN fibers prepared at 900 °C, 1100 °C and 1400 °C, respectively. Low-magnification TEM image of 900 °C sample (Fig. 4a) indicates that the product contains of one-dimensional microfibers with uniform diameters. Numerous bright spots with size of ~15 nm can be observed on the microfibers, indicating that there are many holes existing inside the BN fibers. High resolution TEM (HRTEM) images (Fig. 4b and c) indicate the 900 °C sample consists of many amorphous phases as well as some non-continuous stacking layers, showing poor crystallization. The *d* spacing of the stacking layers is ~0.34 nm, quite close to the (002) interplanar distance of layered *h*BN, suggesting *h*BN phase has been formed after the calcination of M·2B precursor at 900 °C. Nevertheless, enlarged HRTEM image (Fig. 4c) clearly shows that the microfiber contains of only 2 ~ 4 *h*BN stacking layers with small sizes, indicating a large number of defects and impurities exist in the BN fibers.

Figure 4d–f show TEM images of 1100 °C sample. The product shows similar porous microfiber structure with that of 900 °C sample (Fig. 4d). From HRTEM images (Fig. 4e and f) we can find that the BN microfiber after 1100 °C calcination possesses better crystallization than 900 °C BN fiber. The amorphous phase almost disappeared. Instead, 2 ~ 5 parallel fringes can be clearly observed, suggesting the size of the stacking BN layers becomes larger.

TEM images of BN sample prepared at 1400 °C are shown in Fig. 4g–i. The relatively uniform contrast (without bright spots) in Fig. 4g implies that the pores existing in the fibers may have collapsed after the high temperature calcination. In addition, with the increasing crystallization of BN at high temperature, the diffraction contrast should contribute strongly to its TEM images. As a result, the mass-thickness contrast caused by some small pores inside of the specimen could be glossed over. The HRTEM images (Fig. 4h and i) show that the BN fiber contains of many grains with ~10 parallel fringes. This provides evidence that the BN fibers obtained under 1400 °C possess much higher crystallization compared with 900 °C and 1100 °C samples.

**Figure 5:**
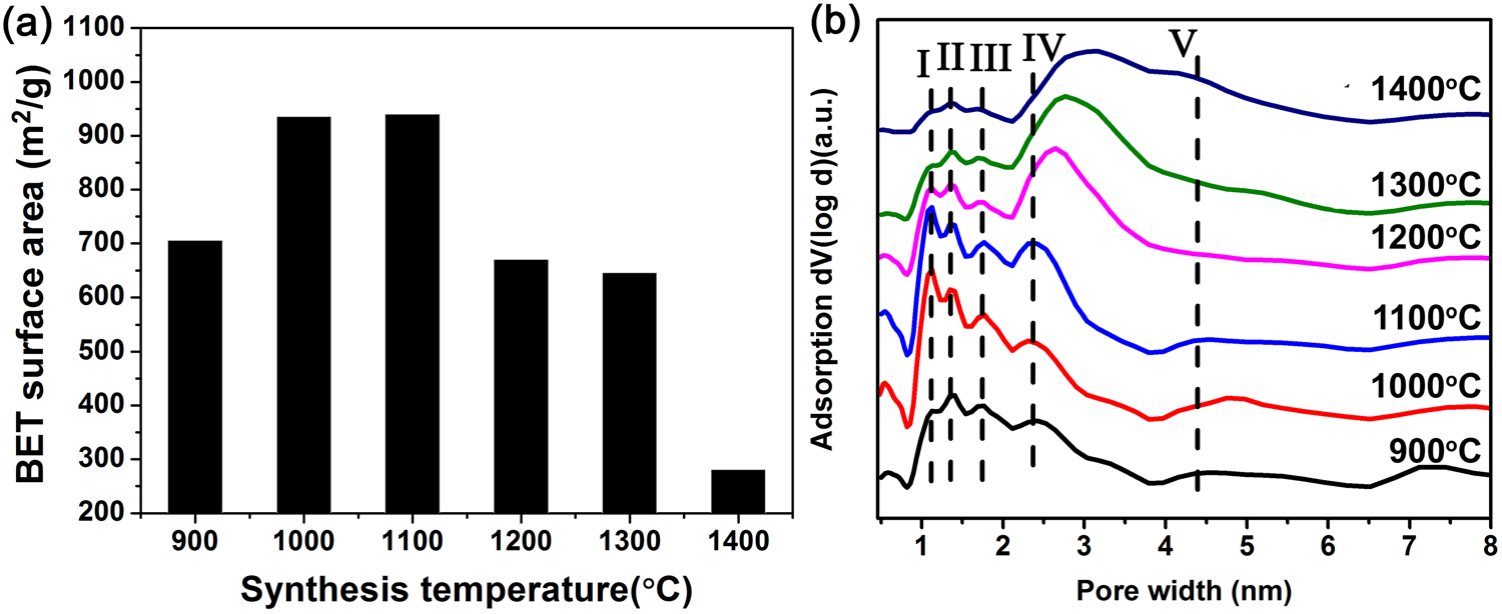
(**a**) The BET specific surface areas of porous BN prepared at different calcination temperatures. (**b**) The corresponding pore size distributions of the samples.

Figure 5a shows the results of Brunauer-Emmett-Teller (BET) specific surface areas of all products prepared at different calcination temperatures. With an increase of calcination temperature, the specific surface area increases at first and then decreases. The 1100 °C-sample possesses the largest specific surface area of 939.7 m^2^/g among all of the products. BN microfibers prepared at low temperature (900 °C) have a poor crystallization with many defects and impurities, thus possess relatively low specific surface area (705.4 m^2^/g). However, the porous structure in BN fibers could be collapsed after very high temperature (1400 °C) calcination, leading to a great decrease of specific surface area (280.5 m^2^/g). This result is in an excellent agreement with our TEM observations. Figure 5b displays the pore size distribution (PSD) of the BN samples calculated by a Non-Local Density Functional Theory (NLDFT) method. All the samples contain of micropores (<2 nm) and mesopores, with main pore widths of I-1.126 nm, II-1.348 nm, III-1.747 nm, IV-2.370 nm and V-4.394 nm. The volumes of micropores in samples synthesized at lower temperatures (900, 1000, 1100 °C) are larger than those obtained at higher temperatures (1200, 1300, 1400 °C). In particular, the 1100 °C-sample has the largest micropore volume.

**Figure 6:**
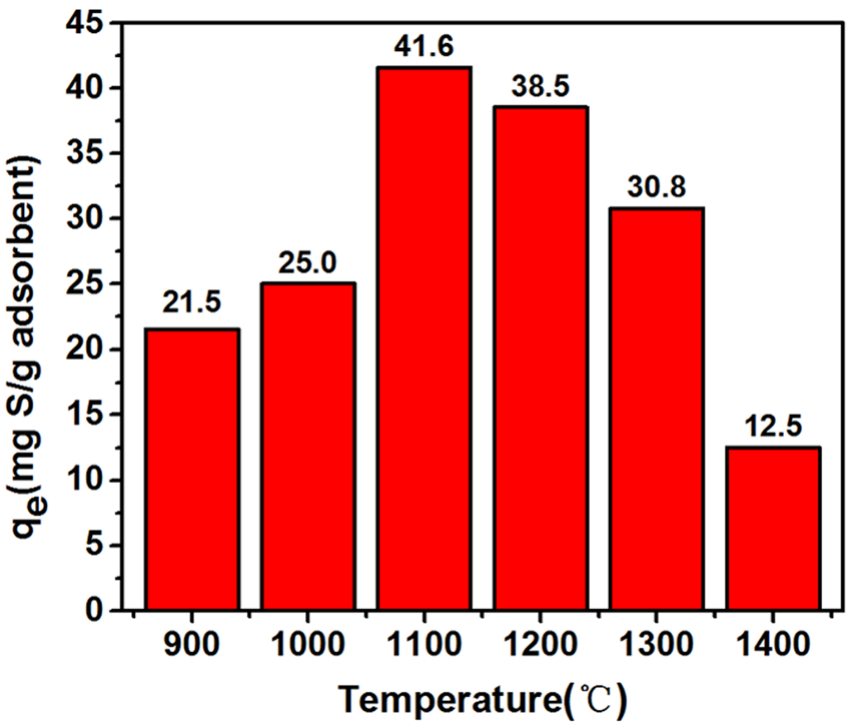
The adsorption capacity of porous BN microfibers prepared at different calcination temperatures.

The as-prepared BN microfibers with high specific surface areas and large pore volumes can be used as valuable adsorbent for desulfurization of model oil. DBT was used as the sulfocompound solute in the model oil with an initial sulfur concentration of 500 mg/L. 50 mg of the as-prepared porous BN fibers was added into the model oil as the adsorbent. The measured adsorption capacities of different BN samples are shown in Fig. 6. Among all of the porous BN products, the 1100 °C-sample exhibits the highest adsorption capacity (41.59 mg S g^−1^), while the 1400 °C-sample shows the lowest adsorption capacity (12.53 mg S g^−1^). We believe that the adsorption capacity of porous BN should be related to three factors: the specific surface areas, the micropore volumes, as well as the surface functional groups. The sample with high specific surface area would supply multiple active adsorption sites, which is beneficial for their adsorption performance. Moreover, since the size of the DBT molecule is as small as ~0.4 nm, porous BN with larger micropore volumes would exhibit stronger adsorption capacity. Besides, the organic surface groups and structural defects in porous BN may also offer great binding sites for the adsorption of DBT.

**Figure 7:**
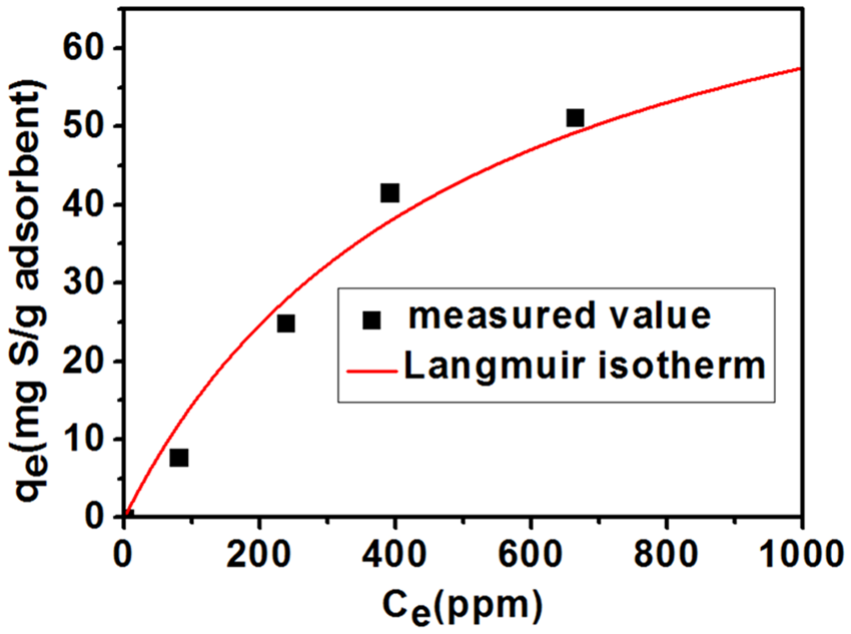
Langmuir isotherm of DBT adsorption porous BN fibers.

Then we studied the adsorption isotherm of DBT on porous BN (1100 °C-sample), as shown in Fig. 7 and Table 1. The theoretical adsorption capacity was obtained via Langmuir equilibrium adsorption isotherm model. The maximum adsorption capacity could reach as high as 86 mg S g^−1^. It worth noting that the high adsorption capacity of the as-prepared porous BN microfibers for DBT (measured value for 41.59 mg S g^−1^ and theoretical value for 86 mg S g^−1^) can not only surpass those of transitional adsorbents such as zeolite (10.9 mg S g^−1^)^34^, mesoporous silica (3 mg S g^−1^)^35^, activated alumina (16.6 mg S g^−1^)^36^, metal-organic frameworks (MOFs) (12 mg S g^−1^)^37^, activated carbon (10.39 mg S g^−1^)^38^, but also rival those of many newly reported adsorbents, i.e. C-doped BN (35.2 mg S g^−1^)^26^, suggesting an excellent adsorptive desulfurization performance of the porous BN microfibers.

**Table 1:** Langmuir constants for the adsorption of DBT on porous BN microfibers.

Langmuir isotherm			
Sample	q_m_(mg S g^−1^ adsorbent)	K(L mg^−1^)	R^2^
BN-1100 °C	86.10	2.01 × 10^−3^	0.98246

**Figure 8:**
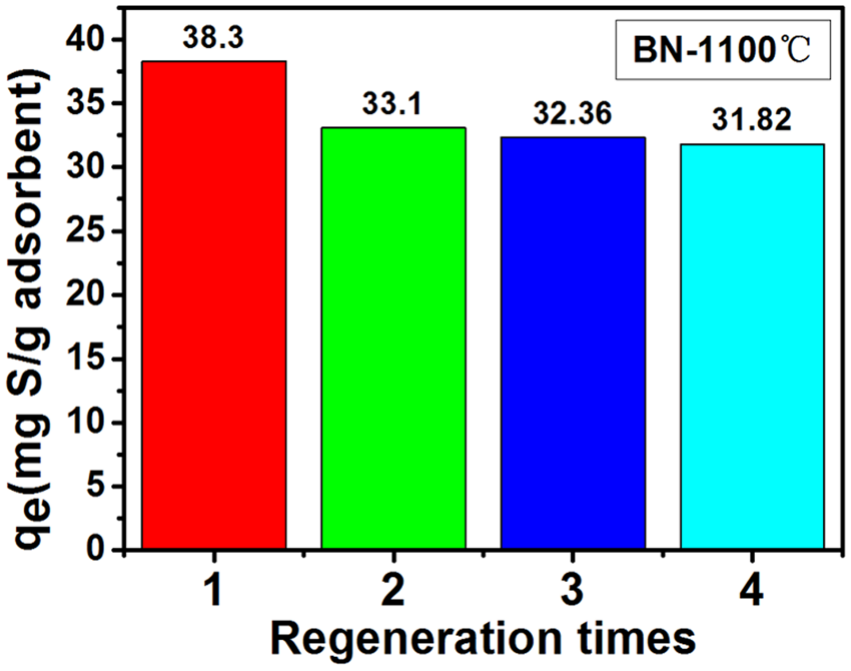
The adsorption capacity of porous BN fibers obtained after different regeneration times.

It is well known that regeneration ability of adsorbent is of great important for their feasible applications. Considering the high thermal stability of BN, a simple heat treatment method has been applied (the sample was heated at 700 °C for 1 h in air) for the regeneration of porous BN microfibers (1100 °C-sample). As shown in Fig. 8, the adsorption capacities are 38.30, 33.10, 32.36 and 31.82 mg S per gram adsorbent for the first run to the fourth run, respectively. The degradation of the adsorption capability is only 23.5% after 4 times regeneration. As comparison, the degradations are 28% for activated carbon after 4 times regeneration, 33.9% for Zn-exchanged NaY zeolites after 2 times regeneration, about 10% for SBA-15/Cu(I) adsorbents after 1 times regeneration and 76.2% for metal organic frameworks after 4 times regeneration, respectively^39–42^. The slight decrease of adsorption capacity is due to the residue after regeneration forming in the channels and blocking adsorption sites.

Compared to the traditional adsorbents, porous BN microfibers hold two advantages for desulfurization application, the very large first-time adsorption capacity and the great regeneration ability. The advantages could greatly reduce the usage and consumption of the adsorbents. From this viewpoint, porous BN microfibers should be promising and economical as adsorbents for desulfurization applications. Nevertheless, for real applications, more research is needed to assess the adsorption in column, affinity toward non-S compounds and the adsorption of S compounds in commercial fuels^43^.

## Conclusions

In summary, we have developed porous BN microfibers as an adsorbent for the desulfurization of model oil. Controllable synthesis of porous BN has been achieved through changing the different calcination temperatures. Detailed XRD, FTIR, Raman and TEM analysis confirm that with an increasing of calcination temperature from 900 °C to 1400 °C, porous BN microfibers display gradually higher crystallization. A moderate calcination temperature (1100 °C) results in the preparation of porous BN with highest specific surface area (939.7 m^2^/g) and largest micropore volume. The as-prepared porous BN microfibers exhibit valuable sulfur adsorption capacity and selectivity for the removal of DBT in the model oil. Especially, the porous BN sample synthesized at 1100 °C displays very high adsorption capacity for DBT (measured value for 41.59 mg S g^−1^ and theoretical value for 86 mg S g^−1^). Such value is superior to the traditional adsorbents such as zeolite, mesoporous silica and activated carbon, and also comparable to some newly reported desulfurization adsorbent, i.e. C-doped BN, suggesting an excellent adsorptive desulfurization performance.

## Methods

### Material synthesis

The H_3_BO_3_ (AR) and C_3_N_6_H_6_ (AR) were used as initial materials to synthesize porous BN fibers. A mixture of H_3_BO_3_ and C_3_N_6_H_6_ with molar ratio of 3:1 was firstly dropped into 500 mL distilled water, which was kept at 98 °C. As the mixture was completely dissolved, the solution was naturally cooled to room temperature to get a white precipitation i.e. C_3_N_6_H_6_·2H_3_BO_3_ (M·2B). After dried at 90 °C for 12 h, the white precipitation, was transferred into a tube furnace and heated at different calcination temperatures (900, 1000, 1100, 1200, 1300 and 1400 °C) in a flow of N_2_ (flow rate 100 ml/min). A series of porous BN microfibers were obtained via decomposition of the precipitation at different temperatures.

### Characterization

The structure of the samples was examined using X-ray powder diffraction (XRD, BRUKER D8 FOCUS) analysis. Fourier transformer infrared (FTIR) spectra were recorded on a Nicolet 7100 spectrophotometer at room temperature. Raman spectra were recorded on a LabRAM HR800 equipped with a 532 nm excitation. Transmission electron microscopy (TEM) analysis was performed on a JEM-2010FEF electron microscope (JEOL, Japan) with an acceleration voltage of 200 kV. The nitrogen physisorption isotherms were measured at −196 °C on an AutoSorb iQ-C TCD analyzer. Prior to the measurement, the samples were activated in a vacuum at 300 °C for 3 h. The Brunauer-Emmett-Teller (BET) specific surface area and pore volume were calculated from the nitrogen adsorption data in the relative pressure ranging from 0.01 to 0.3. The regeneration temperature of the products was tested by simultaneous thermal analysis (SDTQ600).

### Desulfurization tests

Firstly, model oil with different sulfur concentrations (100 mg/L, 300 mg/L, 500 mg/L, 800 mg/L) were prepared using DBT and n-octane as the sulfocompound solute and solvent, respectively. Then 0.05 g of porous BN was added into 20 mL of the as-prepared model oil in an ultrasonic equipment at 25 °C for 30 min (work frequency 40 kHz). Subsequently, the mixture was transferred into a constant temperature bath oscillator. After vibration at 150 rpm for 16 h, the mixed solution was centrifugalized at 10000 rpm for 30 min to obtain the adsorbent and model oil separately.

### Inspection method

After the adsorption of model oil, residual sulfur concentration was analyzed by a Micro coulomb sulfur analyzer with standard material for sulfur determination (LTD WKL-3000).

The adsorbed amount (q_e_) of the sulfur compounds was calculated by using the following Eq. (1)

1 $${{\rm{q}}}_{{\rm{e}}}=({{\rm{C}}}_{0}-{{\rm{C}}}_{e}){\rm{V}}/{\rm{m}}$$

where C_0_ and C_e_ (mg L^−1^) were the initial sulfur concentration and the equilibrium sulfur concentration, and V (L) was the volume of the model oil, and m (g) was the mass of the adsorbent, respectively.

Adsorbate distribution between the adsorbent and the medium was described by equilibrium adsorption isotherm model^44^. The adsorption isotherms were employed to analyze the experimental data. The linear form of the Langmuir model was following Eq. (2)

2 $${{\rm{q}}}_{{\rm{e}}}={{\rm{q}}}_{{\rm{m}}}{{\rm{KC}}}_{{\rm{e}}}/(1+{{\rm{KC}}}_{{\rm{e}}})$$

where C_e_ was the equilibrium concentration DBT (mg L^−1^), q_e_ was the amount of sulfur adsorbed at equilibrium (mg L^−1^), q_m_ was the theoretical maximum adsorption capacity (mg L^−1^), and K was the Langmuir isotherm constant (mg L^−1^).

### Regeneration test

The regeneration of BN fibers was conducted by a heat treatment method in air. After the centrifugal treatment, the collected adsorbent was dried at 90 °C, then placed into a muffle furnace and heated at 700 °C for 1 h. Subsequently, the adsorbent was reused for the adsorption experiments.

### Data availability statement

The materials, data and associated protocols are promptly available to readers without undue qualifications in material transfer agreements.

## Electronic supplementary material

Supporting Information
