# Supplementary material for: Desulfurization of Model Oil by Selective Adsorption over Porous Boron Nitride Fibers with Tailored Microstructures
Source: Sci Rep. 2017 Jun 12;7:3297. doi: 10.1038/s41598-017-03600-4 (PMC5468236; doi:10.1038/s41598-017-03600-4)
Supplement: Supplementary file 1 — Supporting Information [file 41598_2017_3600_MOESM1_ESM.pdf]

Supporting Information

Desulfurization of Model Oil by Selective Adsorption over Porous Boron Nitride Fibers with Tailored Microstructures

Zhiyi Yan[1, 2], Jing Lin[1, 2, *], Xiaohai Yuan[1, 2], Tao Song[1, 2], Chao Yu[1, 2], Zhenya Liu[1, 2], Xin He[1, 2], Jianli Liang[1, 2],Chengchun Tang[1, 2], and Yang Huang[1, 2, *]

[1]School of Materials Science and Engineering, Hebei University of Technology, Tianjin 300130, P. R. China [2]Hebei Key Laboratory of Boron Nitride Micro and Nano Materials, Hebei University of Technology, Tianjin 300130, P. R. China

*Phone: +86-22-60202660; fax: +86-22-60202660; E-mail: linjing@hebut.edu.cn (J. L.); huangyang@hebut.edu.cn (Y. H.)

Table S1. The yield of BN products prepared under different annealing temperatures

Annealing temperatures	The amount of M·2B precursor (g)	Theoretical yield of BN, ($Y_{Theoretical}$, g)*	Actual yield of BN, (Y_{Actual}, g)	Actual yield of BN per gram M·2B (g)	Percentage yield, (%Y, %)**
900°C	5.0000	0.9935	1.0777	0.2155	108.48
1000°C	5.0000	0.9935	1.0675	0.2135	107.45
1100°C	5.0000	0.9935	1.0346	0.2069	104.14
1200°C	5.0000	0.9935	1.0247	0.2049	103.14
1300°C	5.0000	0.9935	1.0216	0.2043	102.83
1400°C	5.0000	0.9935	0.9570	0.1914	96.33

* Theoretical yield ($Y_{Theoretical}$) is calculated according to the following reaction:

$2H_3BO_3 + C_3N_6H_6 \rightarrow C_3N_6H_6 \cdot 2H_3BO_3 \rightarrow 2BN + 3H_2O + 3CO + 2NH_3 + N_2$

** Percentage yield (%Y) is calculated as $\%Y = \frac{Y_{Actual}}{Y_{Theoretical}} \times 100\%$.

As shown in Table S1, the percentage yields of the products obtained under 900-1300 °C are a little higher than 100%, which could be attributed to the adsorption of water and/or gases existing in the atmosphere. Residue impurities may also exist in the products. The yield of the product obtained under 1400 °C is 96.33%.

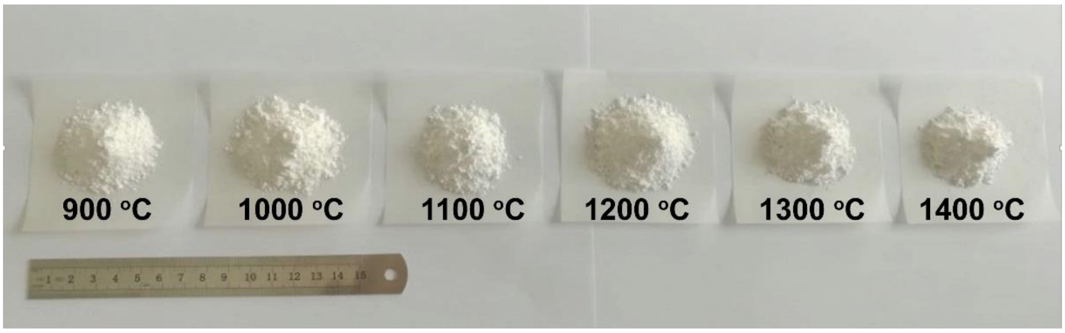

Figure S1. Digital image of BN powders obtained under different annealing temperature. Each pile was obtained from 5 g M-2B precursors.

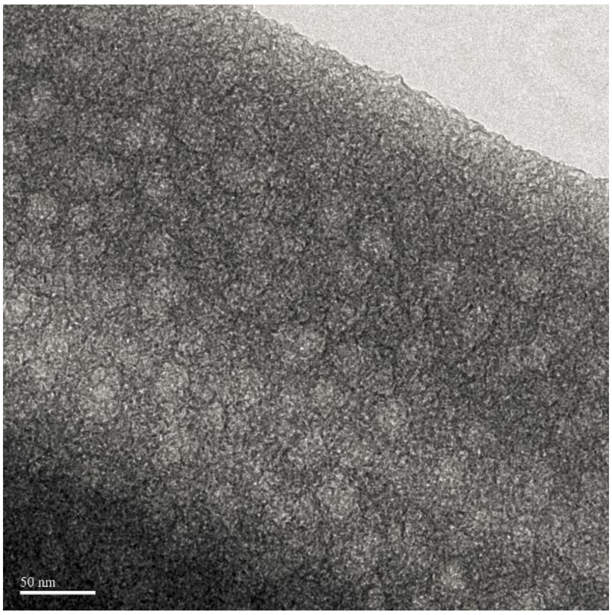

Figure S2. Another TEM image which clearly shows the hollow pores of the BN microfibers obtained under 1100 ℃ calcination
